# Exploring Carbohydrates for Therapeutics: A Review on Future Directions

**DOI:** 10.3389/fphar.2021.756724

**Published:** 2021-11-16

**Authors:** Jie Wang, Yukun Zhang, Qi Lu, Dongming Xing, Renshuai Zhang

**Affiliations:** ^1^ The Affiliated Hospital of Qingdao University, Qingdao University, Qingdao, China; ^2^ Cancer Institute, Qingdao University, Qingdao, China; ^3^ School of Life Sciences, Tsinghua University, Beijing, China

**Keywords:** carbohydrate, drug discovery, drug design, conjugates, glyconanomaterials

## Abstract

Carbohydrates are important components of foods and essential biomolecules performing various biological functions in living systems. A variety of biological activities besides providing fuel have been explored and reported for carbohydrates. Some carbohydrates have been approved for the treatment of various diseases; however, carbohydrate-containing drugs represent only a small portion of all of the drugs on the market. This review summarizes several potential development directions of carbohydrate-containing therapeutics, with the hope of promoting the application of carbohydrates in drug development.

## Introduction

**FIGURE 1:**
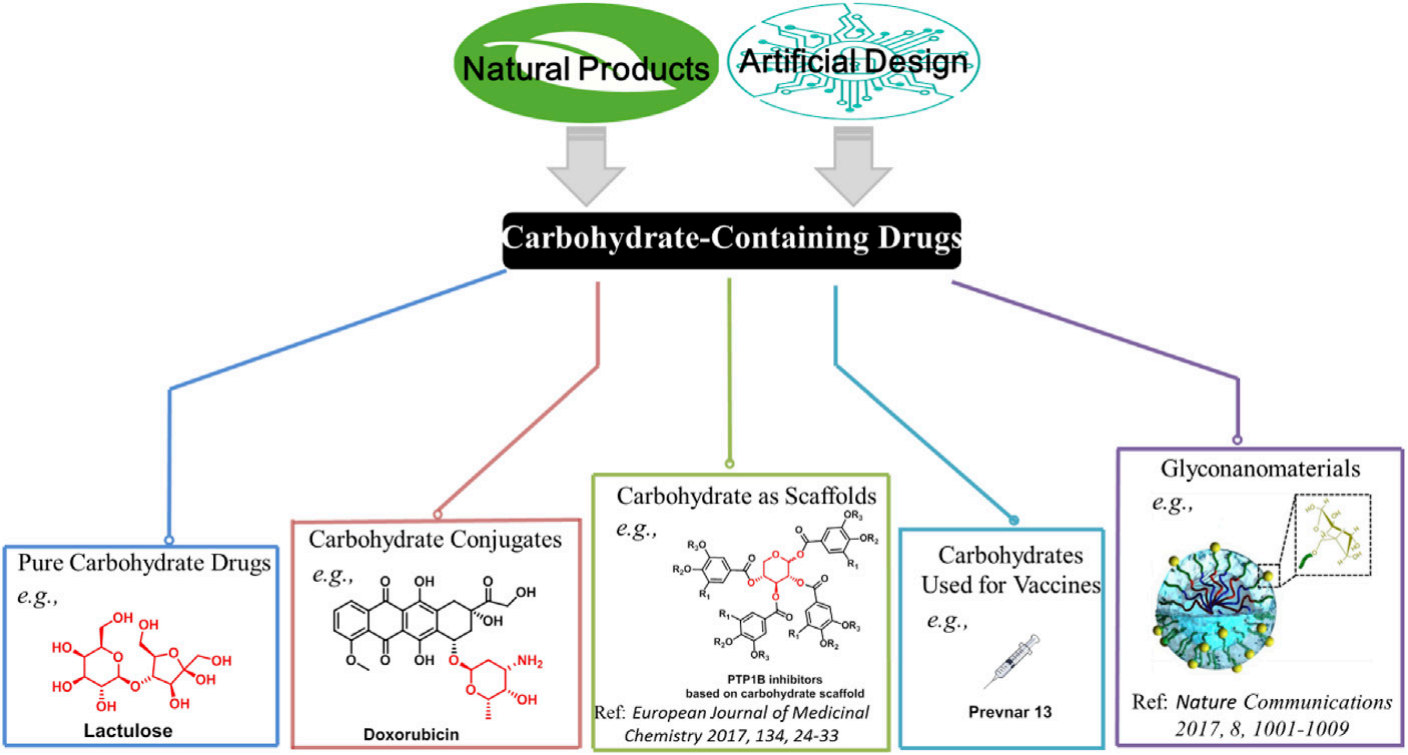
Potential directions in the development of carbohydrate-containing drugs. Representative marketed drugs (or bioactive molecules reported) are displayed.

Carbohydrates are ubiquitously present in a wide range of plants, animals, and microorganisms. Their irreplaceable biological roles have been well established. To date, a large number of carbohydrate-containing drugs have been approved worldwide (Jiang et al., 2021). However, the development of carbohydrate-containing drugs seems to have slowed down in recent years. Of more than 200 drugs that have been approved during 2015–2020, only nine are small-molecule carbohydrate-containing drugs (Bhutani et al., 2021). This mini-review provides a summary and our opinion on the future of carbohydrate-containing drugs. Carbohydrates have three typical characteristics: high density of functional groups (e.g., hydroxyl), diversity of structures based on different configuration, and ideal biocompatibility as they are ubiquitous in the body. It is crucial to harness the intrinsic properties of carbohydrates in order to develop carbohydrate-containing therapeutics. Overall, five potential directions need to be focused on, namely, pure carbohydrate drugs, carbohydrate conjugates, carbohydrate scaffolds, carbohydrates vaccines, and glyconanomaterials (Figure 1).

### Pure Carbohydrate Drugs

**FIGURE 2:**
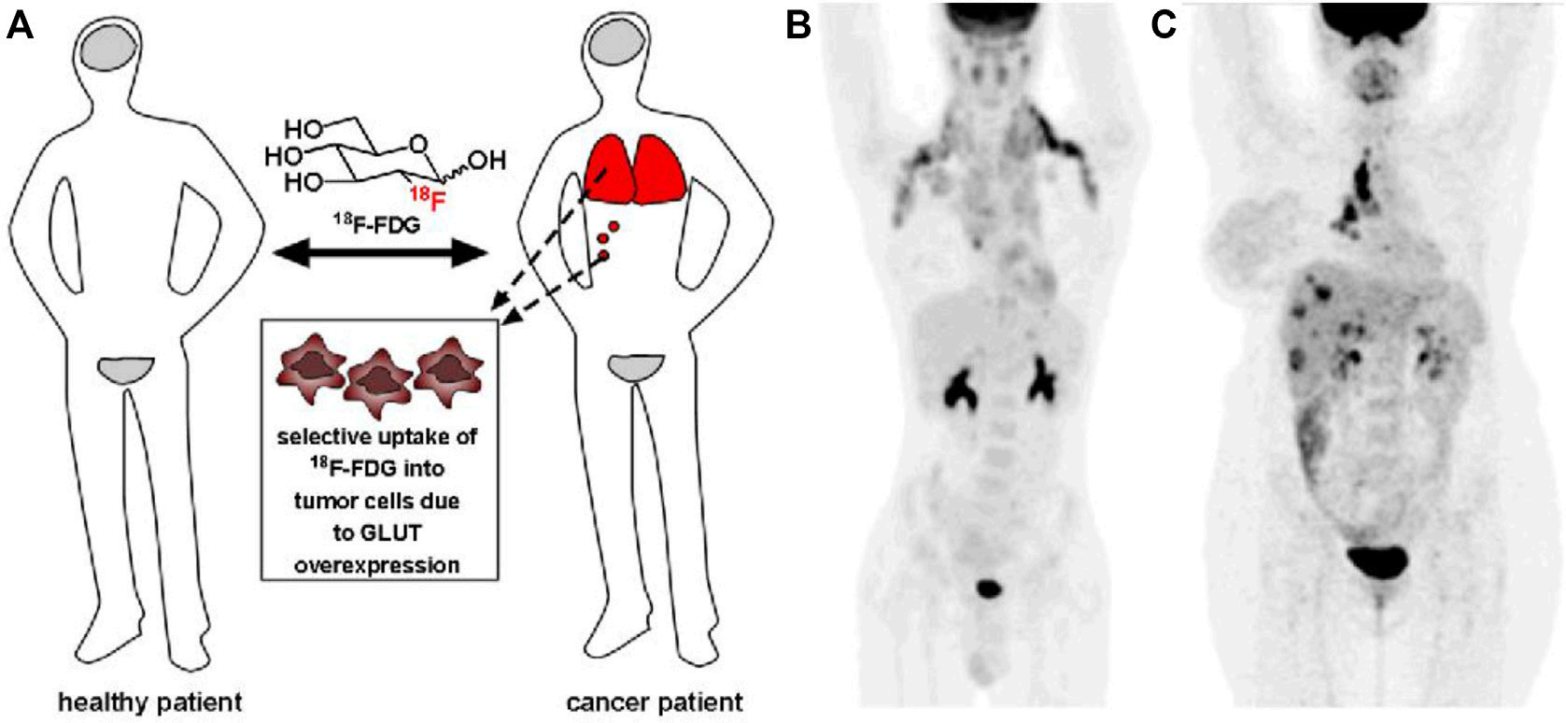
**(A)** Since ^18^F-FDG is preferentially uptaken by tumors, it is used to identify tumor sites and metastases (red). **(B)** ^18^F-FDG positron emission tomography (PET) imaging of a patient with metastatic Hodgkin lymphoma. **(C)** ^18^F-FDG PET imaging of a patient with metastatic breast cancer. Reprinted with permission from Calvaresi and Hergenrother (2013). Copyright 2013 Royal Society of Chemistry.

Carbohydrates present many advantages in drug screening, such as low cost, abundance, high density of functional groups, and diversity of molecular structures. However, in clinical practice, it is rare to directly use carbohydrates as drugs or carbohydrates as the main body of drugs. Monosaccharides are ubiquitous in our body, and thus, they are difficult to directly use as drugs. However, some decorated monosaccharides have been approved for the treatment of specific diseases by mimicking functions of monosaccharides. ^18^F-fluorodeoxyglucose (^18^F-FDG) injection is a typical example (Figure 2). ^18^F-FDG is a radioactive 2-deoxy-2-[^18^F] fluoro-d-glucose that has been used for the diagnosis of cancer in conjunction with positron emission tomography (Ben-Haim and Ell, 2009). Based on the fact that cancerous tissues take up glucose at a higher rate than most normal tissues, ^18^F-FDG is preferentially uptaken by tumor cells, thus allowing clinicians to identify sites of tumors and metastases, as well as to stage cancer and monitor response to treatment.

**FIGURE 3:**
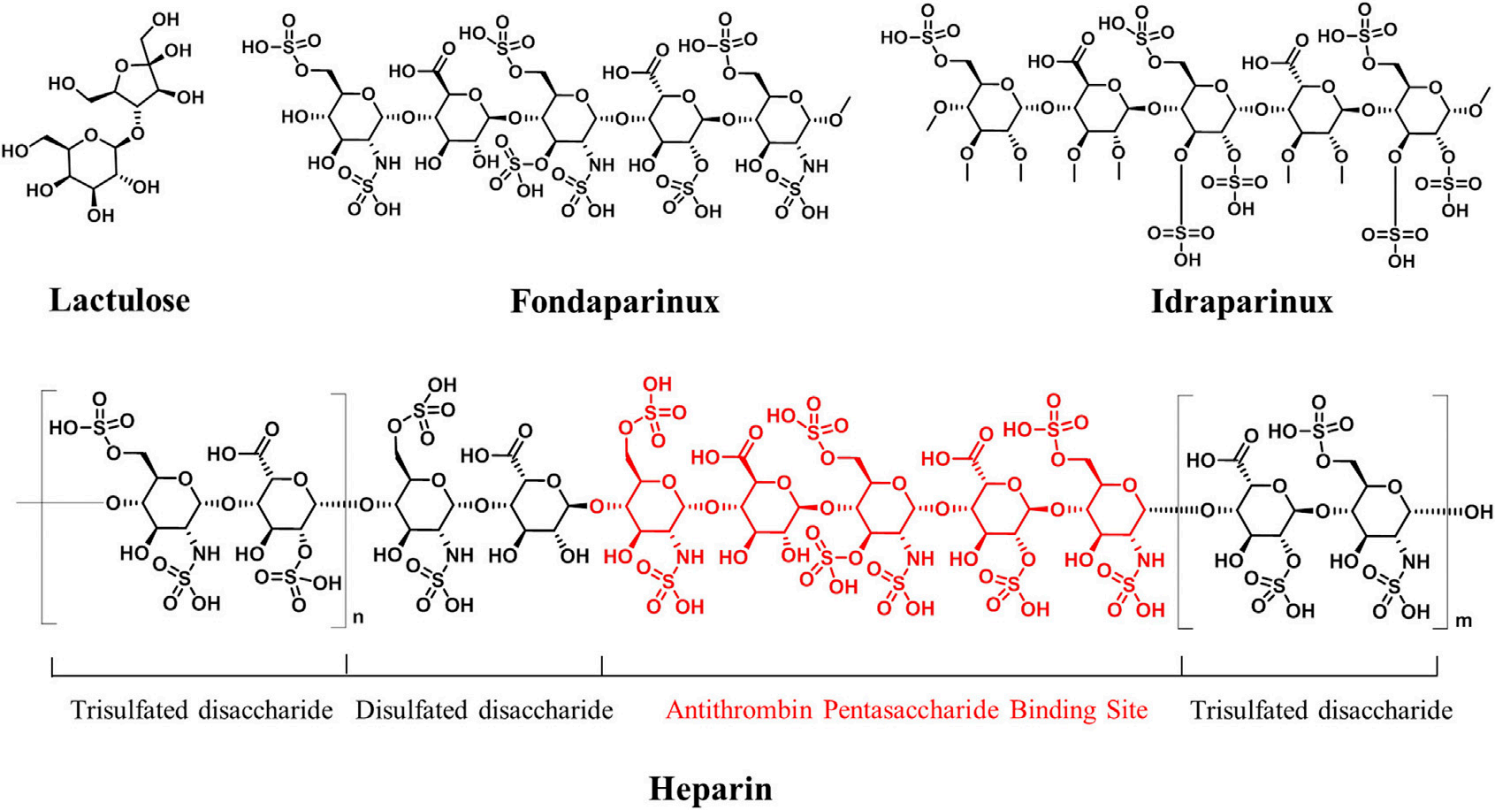
Structures of partly pure carbohydrate drugs. Antithrombin pentasaccharide units of heparin are marked in red.

Oligosaccharides and polysaccharides show low lipophilicity due to the presence of multiple hydroxyl groups. Typically, the less lipophilic a drug, the worse is its absorption following oral administration (Witczak, 2006). Therefore, they are usually designed for the treatment of the gastrointestinal tract diseases, where less absorption is acceptable or necessary. Lactulose (Figure 3), a disaccharide, is a typical example, which undergoes minimal gastrointestinal tract absorption and being broken into organic acids by saccharolytic bacteria and thus enhances intraluminal gas formation and facilitates bowel movements. Therefore, it has been used for the treatment of chronic constipation (Bae and Kim, 2020). In addition, intravenous administration is also an option for this type of therapeutic agent with high polarity. Heparin (Figure 3) is a sulfated polysaccharide isolated from animal organs, and it has been used clinically as an intravenously injected antithrombotic agent for decades (Qiu et al., 2021). However, it is a highly heterogeneous mixture of polysaccharides and is associated with severe side effects. After the structure–function relationship of heparin was established using synthetic oligosaccharides, some antithrombotic agents with definite single structures were developed, such as fondaparinux and idraparinux (Figure 3) (Dey et al., 2019; Bugg et al., 2011). Idraparinux is a fully synthetic analog of the pentasaccharidic domain of heparin, which contains *O*-sulfation and *O*-methyl functionalities instead of *N*-sulfation and free hydroxyl groups. It has a chemically defined structure, and it is also an analog of fondaparinux. Compared with fondaparinux, idraparinux shows a higher anti-Xa activity and longer half-life, and it has been in a phase III clinical trial for the treatment of patients with atrial fibrillation and venous thromboembolic events. Recently, a seaweed-derived oligosaccharide, GV-971, has been approved in China for the treatment of Alzheimer disease (Wang et al., 2019). GV-971 is a heterogeneous mixture of acidic linear oligosaccharides ranging from dimers to decamers. A study recently published by Geng et al. showed that GV-971 could decrease Aβ-related pathologies by reconditioning the gut microbiota. With the establishment of the structure–function relationship of GV971, oligosaccharides with definite single structures may open new possibilities in the therapeutic field of Alzheimer disease.

### Carbohydrate Conjugates

**FIGURE 4:**
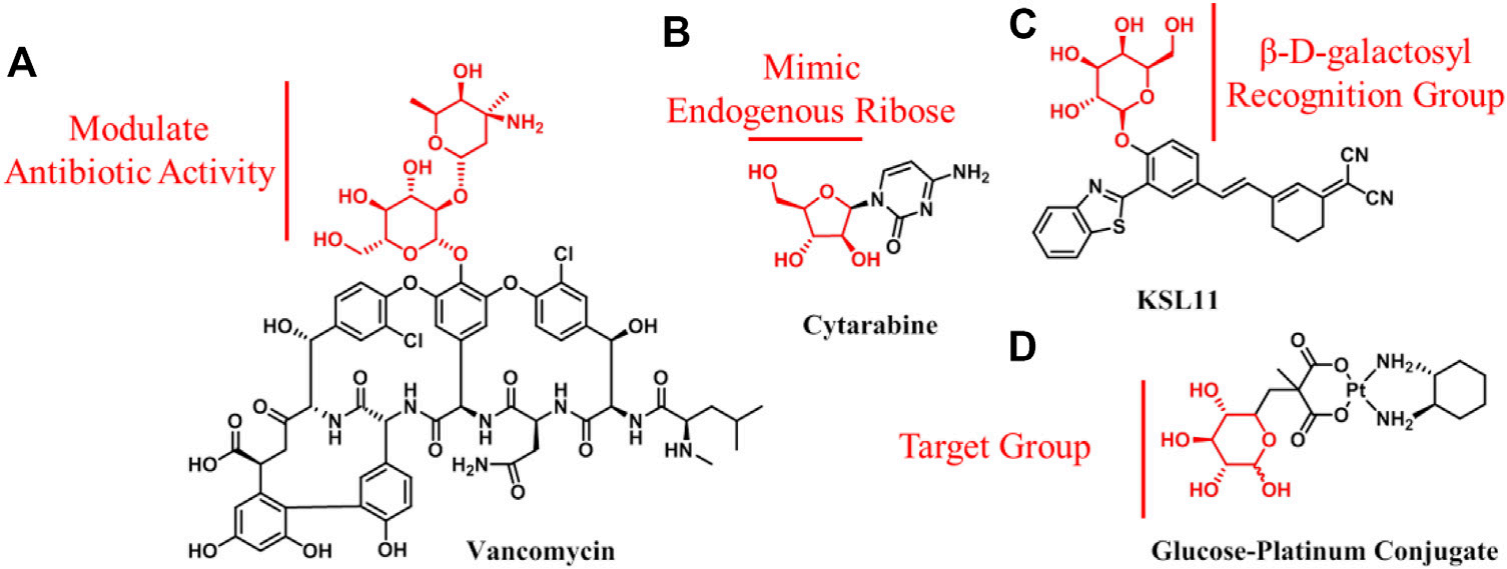
Structures of carbohydrate conjugates: **(A)** Vancomycin used as an anti-infection agent; **(B)** nucleoside analog, cytarabine; **(C)** fluorescence probe of human senescence-associated β-galactosidase with potential diagnostic application; **(D)** carbohydrate, as a target group, is used to design anticancer agents.

Carbohydrate conjugates refer to carbohydrates that are used in the attachment of drugs. In this case, the carbohydrate molecule itself is not the main body of drugs; rather, it is a functional group utilized to increase their bioactivity, improve physical and chemical properties, or achieve targeting. The vast majority of the drugs containing carbohydrates that are already on the market fall into this category. This is not surprising, given that the high polarity and multifunctional properties of carbohydrates make them ideal additions to improve drug properties. However, it is noteworthy that either these approved drugs have originated from natural products containing carbohydrate molecules (e.g., antibiotics and vancomycin; Figure 4A), or they have been designed based on the key components containing carbohydrates in the body (e.g., nucleoside analogs and cytarabine; Figure 4B) (Jordheim et al., 2013; Yilmaz and Ozcengiz, 2017). Apart from these two aspects, carbohydrates have much more potential for exploitation in drug design. For example, a number of carbohydrate-containing probes with potential diagnostic applications have been reported (e.g., KSL11; Figure 4C); they could be used to detect ions, small molecules, and enzymes (Yang et al., 2010; Li et al., 2011; Longxia Li, 2015; Li et al., 2020). Furthermore, carbohydrates (glucose or other glucose transporter substrates) have been conjugated to cytotoxins or anticancer therapeutics for the specific targeting and treatment of cancer (Calvaresi and Hergenrother, 2013). The rationale behind this strategy is that glucose transporters and glycolytic enzymes are widely overexpressed in cancer tissues, which highly correlates with poor cancer prognosis, thus making them attractive therapeutic targets for achieving anticancer drug targeting (Treekoon et al., 2021). The wide application of ^18^F-FDG injection in diagnosis and staging of many types of cancer provides the strongest support to this theory. Cancers clinically staged using ^18^F-FDG imaging may be good candidates for glycoconjugate targeting. According to Bensinger et al., they include lung, breast, colorectal, and endometrial carcinomas, as well as bone and soft tissue sarcomas and Hodgkin and non-Hodgkin lymphomas (Bensinger and Christofk, 2012). Actually, carbohydrate-conjugated anticancer active molecules for targeting therapy have attracted great interest and grown markedly in recent years. Several clinical trials in several countries over the past decades have been conducted on glufosfamide, a trailblazer for glycoconjugated anticancer agents (Briasoulis et al., 2003; Ciuleanu et al., 2009). In addition, some glycoconjugates have shown improved activity and selectivity compared with aglycone *in vitro* and *in vivo* (e.g., glucose–platinum conjugate; Figure 4D) (Mikuni et al., 2008; Miot-Noirault et al., 2011; Patra et al., 2016; Wang et al., 2016; Zhang et al., 2017). However, this drug design strategy has not yet been proven in clinical practice, which somewhat undermines the confidence of researchers in the field. The questions remain as to how to choose the proper coupling position of carbohydrate, what is the exact mechanism of glycoconjugates entry into cells, and how glycoconjugates work *in vivo*.

### Carbohydrate as Scaffolds

**FIGURE 5:**
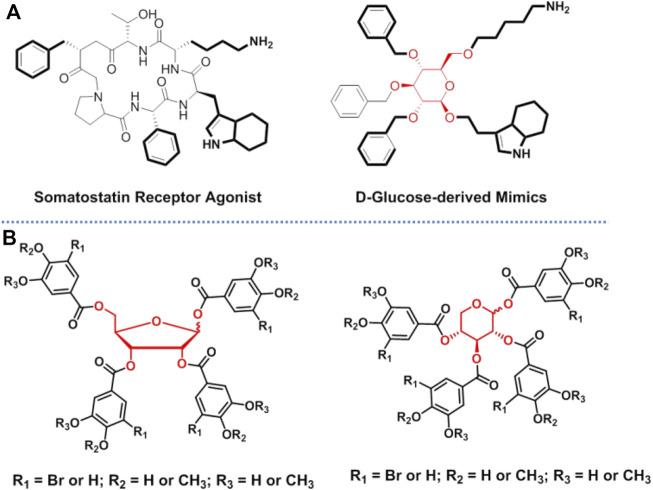
Carbohydrates used as scaffolds in drug discovery: **(A)** glucose was used to mimic the backbone of peptides; and **(B)** carbohydrates were used in the screening of PTP1B inhibitors.

Carbohydrates have high functional group density and diversity of functional group orientations, which makes them excellent scaffolds for designing bioactive compounds by appending desired substituents at selected positions around the sugar ring. This strategy has promising prospects in drug development, and it has widely been used in the design of peptidomimetics. It is known that the instability of the amide backbone is an important limiting factor in the development of peptide drugs. In addition, the amide backbone makes the peptides less permeable to membranes, which leads to their lower bioavailability. Accordingly, using carbohydrates to mimic the backbone of peptides is a promising strategy for increasing the drug ability of peptides. Since Hirschmann et al. first reported that the peptidomimetic based on β-d-glucoside scaffold (Figure 5A) could target somatostatin receptor, several research groups have reported the various applications of peptidomimetics based on carbohydrate scaffolds in different biological fields (Hirschmann et al., 1992; Hirschmann et al., 2009). Relevant studies of peptidomimetics have been reviewed extensively elsewhere (Cipolla et al., 2005; Meutermans et al., 2006; Velter et al., 2006; Cipolla et al., 2010; Tian et al., 2015; Lenci and Trabocchi, 2020). However, the existing examples have also demonstrated the difficulty of designing single carbohydrate scaffold mimetics that maintain the level of bioactivity (and/or selectivity) of the counterparts. This is because it is difficult to ensure that the positions and orientations of the functional groups of the mimetics are exactly the same as those of the counterparts (original ligands). A possible solution is to use carbohydrates as scaffolds to build a diverse library of compounds and to use the library to screen the ideal molecules (Hollingsworth and Wang, 2000; Le et al., 2003; Lenci et al., 2016). The novel strategy has been used to screen inhibitors of protein tyrosine phosphatase 1B (PTP1B) in our group, where ribose and xylose were used as scaffold (Figure 5B) (Zhang et al., 2017). The successful obtainment of a potent and selective PTP1B inhibitor preliminarily proved the feasibility of this strategy. However, in the current research, carbohydrate scaffolds were modified with the same pharmacophore in one molecule, which did reduce the difficulty of the reaction, but also dramatically reduced the diversity of compounds. Presenting different pharmacophores at different substitution sites of the carbohydrate scaffold can greatly increase the diversity of compounds. Thus, based on this research idea, efficient organic synthesis methods that involve the individual sequential protection and deprotection of single hydroxyl group on sugar rings are essential. Advances in organic synthesis in the field of carbohydrate chemistry have accelerated and extended the application of this strategy (Busto, 2016; Ghosh and Kulkarni, 2020). It is expected that as the power of organic synthesis increases, it will be an alternative for drug development to utilize carbohydrates as scaffolds to generate compound libraries that are highly functional and structurally diverse and to further screen bioactive molecules.

### Carbohydrates Used for Vaccines

Carbohydrates have also been used for the development of vaccines, such as carbohydrate-based antimicrobial vaccines and anticancer vaccines (Mettu et al., 2020). The star drug *Prevnar 13* is a typical polysaccharide–protein conjugate vaccine; it was approved by the US Food and Drug Administration in 2010 (Gruber et al., 2012). The rationale behind carbohydrate-based vaccines is based on the theory that carbohydrates could be recognized as antigenic determinants, and they could be specifically recognized by the immune system (Temme et al., 2021). Capsular polysaccharides and lipopolysaccharides are the major constituents of the microbial cell surfaces and can be specifically recognized by the host immune system; thus, they could be exploited as the basis for the design of antibacterial vaccines. Expression of carbohydrates on the cancer cell surface is different from that on the normal cell surface. Abnormal glycosylation in primary tumors closely correlates with the survival rate of cancer patients. These abnormal oligosaccharides or polysaccharides, usually referred to as tumor-associated carbohydrate antigens, are strictly related to the metastasis of tumor cells. Based on the immunogenicity of carbohydrates, the development of carbohydrate-based vaccines provides an attractive option for the treatment of infections and cancers. The main difficulty in carbohydrate vaccine development is the poor immunogenicity due to their inherent T-cell–independent nature. In the response to this class of carbohydrate antigens, no immunological memory is established, and no T-cell responses are induced, which is markedly different from responses to proteins and peptides. One method of overcoming this problem is to conjugate the corresponding carbohydrates to a carrier protein (Galili, 2020). However, we need to solve first how to obtain carbohydrate antigens with sufficient quantity, high purity, and structural integrity. In addition, natural polysaccharides obtained by separation are usually characterized by significant heterogeneity and easy contamination. Several studies have demonstrated that synthetic conjugates show comparable activity to the native polysaccharides linked to the same carrier protein, indicating that the chemically well-defined synthetic oligosaccharide is safer than the natural polysaccharide counterpart. Moreover, fully synthetic oligosaccharide conjugate vaccines have more advantages because they could be designed to incorporate only elements required for a desired immune response, and they could produce chemically well-defined compounds in a reproducible fashion. Therefore, efficient synthetic methods are critical for the development of carbohydrate-based vaccines. Although the construction of oligosaccharides remains a challenging task due to the combined demands of elaborate procedures for glycosyl donor and acceptor preparation and the requirements of regio- and stereo-selectivity in glycoside bond formation, considerable improvements have been made in this field (Zhu and Schmidt, 2009; Ghosh and Kulkarni, 2020; He et al., 2020). It is expected that new and efficient synthetic methods will be developed in the near future, which will give access to a wide range of oligosaccharides and glycoconjugates for vaccine development.

### Glyconanomaterials

**FIGURE 6:**
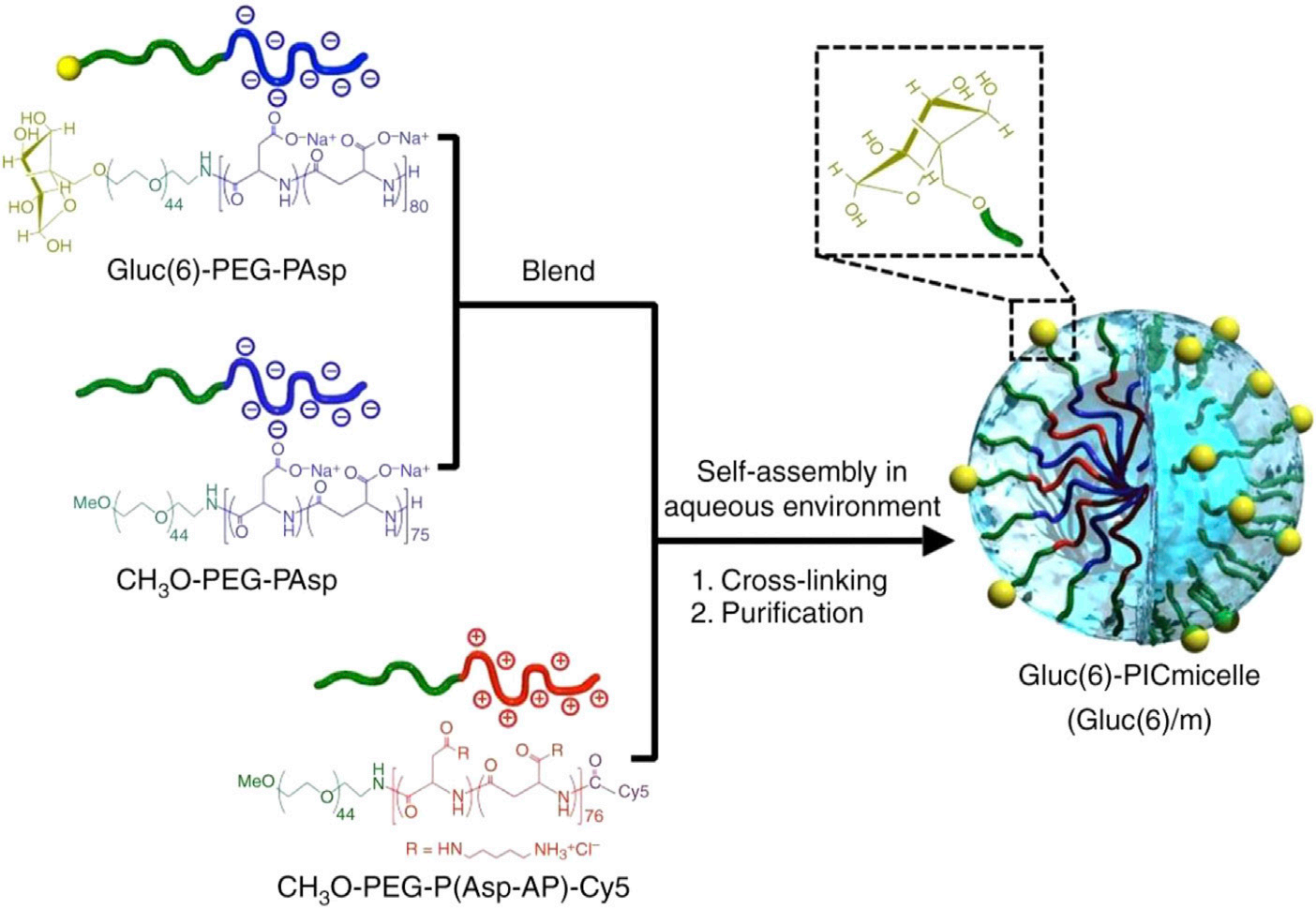
A glucosylated nanocarrier used to deliver drugs able to cross the BBB and reach the brain tissue. Reprinted with permission from Anraku et al. (2017).

Carbohydrates have been conjugated to nanomaterials for biomedical imaging, diagnostics, and therapeutics (Wang et al., 2010; Crucho and Barros, 2019). In addition, some carbohydrate-containing drugs have been used in nanodelivery systems as cargos for enhancing drug efficacy, reducing nonspecific toxicity or improving targeting (Senanayake et al., 2011; Delorme et al., 2020). The combination of glycochemobiology and nanotechnology has provided promising new tools that could be used in imaging of cancer cells, photodynamic therapy, biosensors, and drug targeting (Reichardt et al., 2016). In this section, the application of carbohydrates in the modification of nanomaterials is highlighted. Considering their ubiquitous distribution in tissues and important functions at the cellular level, carbohydrates have been widely used in the functionalization of nanomaterials and have shown unique advantages in the development of nanomedicines. Strategies for the design, preparation, and application of glyconanomaterials have been summarized in many recent reviews (Gorityala et al., 2010; Marradi et al., 2013; Hao et al., 2016; Zhang et al., 2018; Khan et al., 2019). Generally, carbohydrates have three typical advantages in the development of nanomedicine. In addition to increasing water solubility, the main advantages are improving the biocompatibility of nanomaterial and facilitating the improvement of affinity for receptors. In terms of biocompatibility, carbohydrates are an ideal choice to overcome nanomaterial immunogenicity that has limited the use of nanomaterials *in vivo* to a large extent. The rationale behind this advantage is that host-like glycan structures in a form of molecular mimicry could assist some pathogens to evade recognition from the host immune system (Severi et al., 2007). Thus, it is not surprising that carbohydrate-decorated nanoparticles have lower immunogenicity compared with unmodified nanomaterials. As a major nonimmunogenic carbohydrate component, glucose has been used in the design of glyconanoparticles in the study by Frigell et al. (2013). In terms of increasing affinity, the synergy between nanomaterials and carbohydrates showed huge potential advantages. Nanomaterials, a kind of formidable platform, could make the presentation of multiple carbohydrate ligands possible *via* the large surface-to-volume ratio, thereby greatly increasing the affinity of carbohydrates as biofunctional ligands for specific glycan-binding proteins. The study by Reynolds et al. showed that the gold nanoparticle platform displayed a significant cluster glycoside effect for presenting carbohydrate ligands with almost a 3,000-fold increase in binding compared with a monovalent reference probe in free solution (Reynolds et al., 2012). The targeting aggregation capacity of carbohydrates has further been demonstrated in the study of nanocarrier-mediated drug delivery into the brain (Anraku et al., 2017). According to Anraku et al., the nanocarrier with a surface featuring many glucose molecules has the potential for delivering various drugs directly into the brain by crossing the blood–brain barrier (BBB) through taking advantage of a multivalent interaction between multiple glucose molecules and glucose transporters (Figure 6) (Anraku et al., 2017). Further, carbohydrate-decorated nanoparticles could be used as the active therapeutic entities to inhibit pathogen adhesion—the first step to initiate infection. A study showed that 120 monosaccharides that functionalized tridecafullerene exhibited a potent inhibitory effect against cell infection caused by an artificial Ebola virus with half-maximum inhibitory concentrations in the subnanomolar range (Muñoz et al., 2016). More recently, Bhatia et al. reported adaptive flexible sialylated nanogels that could deform and adapt onto the influenza A virus surface via multivalent binding of sialic acid residues with hemagglutinin spike proteins on the virus surface. Based on the multivalent binding strategy, sialylated nanogels could efficiently block the virus adhesion on cells and inhibit the infection at low pM concentrations (Bhatia et al., 2020).

As for carbohydrate-decorated nanomaterials, the key aspects of their performance include the proper display of carbohydrate ligands, the type and length of the spacer linkage, and the ligand density. Receptors have the best affinity for specific carbohydrate molecules; for example, glucose transporter-1 shows higher affinity to glucose compared with other monosaccharides; therefore, the selection of carbohydrate molecules is crucial in the functionalization of nanomaterials. In addition, the effect of linkers on the binding affinity of glyconanoparticles has also been investigated in recent studies (Wang et al., 2010; Richards et al., 2012; Simpson et al., 2016; Malakootikhah et al., 2017). The results showed that the binding affinity increased with the spacer linker length. The longer and more flexible spacer may provide additional spatial freedom and less steric hindrance to the attached ligands for a more efficient association with their binding partners. Regarding the effect of carbohydrate ligand density, it is reasonable that carbohydrate molecules recognize receptors principally through weak interactions such as hydrogen bonding; thus, increasing the carbohydrate ligand density may introduce cluster or multivalency effects, which could significantly enhance the binding affinity. The most representative example is that oligosaccharides usually exhibit higher binding affinity than monosaccharides toward the same lectin receptor. Although the binding affinity could be roughly quantified by technical means already available, such as surface plasmon resonance or isothermal titration calorimetry, in general, it is difficult to control the number of carbohydrate ligands conjugated to relative nanomaterials. Such imprecise preparation methods may result in ambiguities in composition and structure and batch-wise variations of prepared glyconanomaterials, which is one of the important factors limiting carbohydrates’ clinical development.

## Conclusion

Overall, the present review summarized the possible directions of carbohydrate-containing drugs based on the internal characteristics of carbohydrates. As the biological functions of carbohydrates continue to be explored and more novel carbohydrate-containing molecules are artificially designed or obtained from natural products, it is expected that carbohydrates as the treasure house of medicine will bring more surprises to us in the near future.
